# ClC‐2 Contributes to Hypotonicity‐Induced Adrenal Aldosterone Secretion

**DOI:** 10.1111/apha.70168

**Published:** 2026-01-28

**Authors:** Marina Volkert, Hoang An Dinh, Ute I. Scholl, Gabriel Stölting

**Affiliations:** ^1^ Center of Genomic Medicine Berlin Institute of Health at Charité—Universitätsmedizin Berlin Berlin Germany; ^2^ Institute of Translational Physiology Charité—Universitätsmedizin Berlin Berlin Germany; ^3^ Department of Nephrology and Medical Intensive Care Charité—Universitätsmedizin Berlin Berlin Germany

**Keywords:** adrenal gland, aldosterone, chloride, chloride channel, osmoregulation

## Abstract

**Aim:**

The zona glomerulosa (ZG) of the adrenal cortex regulates blood pressure and electrolyte homeostasis through aldosterone production. In ZG cells, potassium and angiotensin II (Ang II) trigger calcium oscillations that drive aldosterone synthesis. Changes in serum osmolality also modulate aldosterone production in a chloride‐dependent fashion, but the involved proteins remain unclear. Because the chloride channel ClC‐2 is activated by hypoosmolality, we investigated its role in ZG osmoregulation.

**Methods:**

We used *Clcn2* knockout (KO) and wild‐type (WT) mice. Explanted adrenal glands were incubated with iso‐ and hypotonic solutions for measurements of aldosterone. Acute adrenal slices were studied using calcium and chloride sensitive fluorescent dyes. We also investigated ClC‐2's systemic importance by inducing a hyponatremic hypoosmolality in mice using desmopressin.

**Results:**

Under hypoosmolar conditions, WT adrenals upregulated aldosterone production in vitro, an effect that was absent in the KO. WT cells responded to hypoosmolality with increased intracellular calcium levels. This response was abrogated in KO cells. Intracellular chloride levels were higher in ZG cells from KO adrenal slices. This suggests that ClC‐2 provides a hypoosmolality‐dependent chloride efflux pathway that is missing in the KO. Systemic hypoosmolality in mice induced by desmopressin did not differentially affect blood aldosterone levels.

**Conclusion:**

ClC‐2 plays a role in the ZG's response to reduced extracellular osmolality through chloride outflow, which likely causes depolarization, voltage‐dependent calcium influx, and aldosterone production. These data advance our understanding of regulators of aldosterone production.

## 1 Introduction

The adrenal glands, paired organs located cranial to the kidneys, consist of the neuroendocrine medulla as well as the steroid‐hormone producing cortex. The cortex comprises distinct functional layers, with the zona glomerulosa (ZG) forming the outermost layer responsible for mineralocorticoid synthesis. The mineralocorticoid aldosterone is required for maintaining salt and water homeostasis, acting primarily via altering water and salt reabsorption in the kidneys and intestine.

ZG cells regulate aldosterone production primarily in response to the extracellular concentrations of angiotensin II (Ang II) and potassium [1, 2]. Ang II triggers membrane depolarization through the closure of background potassium channels, leading to a depolarization of the ZG [3], whereas hyperkalemia directly depolarizes the cell. This facilitates calcium influx via T‐ and L‐type voltage‐gated calcium channels [4], regulating aldosterone synthesis [5].

Mutations in several ion channel genes enhance this depolarization or calcium influx, resulting in an excessive and uncontrolled synthesis of aldosterone in a disease termed primary aldosteronism [6, 7, 8, 9]. Among these, gain‐of‐function variants in the voltage‐gated chloride channel ClC‐2 (encoded by the *CLCN2* gene) were identified in the germline of patients with familial hyperaldosteronism [10, 11] but also as somatic mutations in aldosterone‐producing adenomas [12].

ClC‐2 is a nearly ubiquitously expressed, inwardly rectifying chloride channel [13, 14]. Apart from its role in primary aldosteronism [15], various other pathologies are associated with gain‐ or loss‐of‐function variants, including in brain [16, 17] and heart [18].

Despite the thorough investigations into the effects of pathogenic variants, we know much less about the physiological role of ClC‐2 in mammalian cells and tissues. In murine colonic epithelium, the basolateral channel mediates chloride reabsorption [19, 20]. Its loss leads to degeneration of testicular Sertoli cells [21] as well as of the retinal pigment epithelium [21, 22, 23], although the precise molecular mechanisms remain unclear [24].

ClC‐2 activates upon voltages negative to the equilibrium potential for chloride. ZG cells possess a high resting intracellular chloride concentration of about 70 mmol/L [10]. Activation of ClC‐2 near the resting membrane potential should therefore result in a depolarizing chloride efflux. This mechanism also underlies the known ClC‐2 gain‐of‐function mutations in primary aldosteronism by facilitating voltage‐dependent calcium influx and subsequently aldosterone synthesis [25, 26]. This, however, does not explain the physiological role of wild‐type ClC‐2 in the ZG. We previously hypothesized that its slow hyperpolarization‐dependent activation might mediate the initial depolarization required for voltage oscillations in the ZG [10, 25] but ClC‐2 KO mice show normal aldosterone levels [25, 26], arguing against such a role.

There is a longstanding debate on the physiological role of ClC‐2's activation by hypoosmolality [13, 27], and it has been implicated in osmoregulation with the best examples coming from heterologous expression systems [27] and malaria‐infected cells [28].

Mice exhibit higher isotonicity than humans, which is also strain dependent [29, 30]. Published values for C57BL/6 mice average ~312 mosmol/kg H_2_O, which we chose as isotonic for this study. Given the known chloride‐dependent osmo‐sensitivity of the ZG [31, 32, 33], we set out to examine the involvement of ClC‐2 in hypoosmolality‐induced aldosterone production.

To do so, we investigated the systemic as well as the cellular response in acute slice preparations of murine WT and ClC‐2 KO adrenal glands using chloride and calcium imaging together with an analysis of aldosterone production.

## 2 Results

### 2.1 Hypoosmolality Increases Aldosterone Production in WT but Not *Clcn2* KO Mice

**FIGURE 1:**
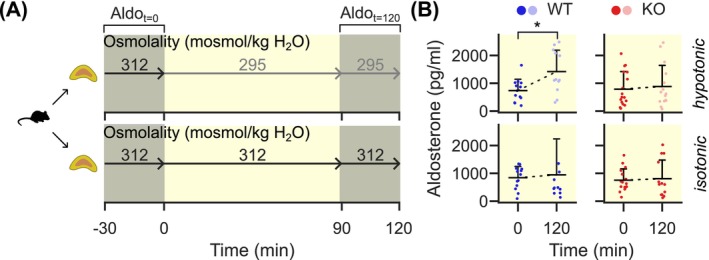
Incubation of whole adrenal glands in vitro demonstrates osmolality‐dependent aldosterone secretion in WT mice. (A) From each mouse, the two glands were separately incubated with continuous supply of 95% O_2_ and 5% CO_2_, as well as 100 pmol/L Ang II and 5 mmol/L K^+^. Initially, both glands were kept under isotonic conditions for 30 min. Then, solutions were exchanged so that one adrenal gland was exposed to a hypotonic solution (295 mosmol/kg H_2_O). The other gland remained as a control with fresh 312 mosmol/kg H_2_O solution. Solutions were again replaced after another 90 min and aldosterone levels determined after another 30 min (total of 120 min). (B) Incubation with a hypotonic solution resulted in an increase in aldosterone production compared to the initial values in WT (blue). This effect was absent in KO mice (median: WT 295 mosmol/kg H_2_O: 1125 pg/mL, 312 mosmol/kg H_2_O: 734 pg/mL, *n* = 12 mice, T = 10, *p* = 0.02, *; KO 295 mosmol/kg H_2_O: 619 pg/mL, 312 mosmol/kg H_2_O: 499 pg/mL, *n* = 15 mice, T = 59, *p* = 0.98; Wilcoxon signed‐rank test). Each dot represents one adrenal gland, horizontal black lines indicate the mean, with the whisker representing the SD (only the positive whisker is shown for clarity).

To test whether extracellular hypotonicity differentially affects aldosterone synthesis in WT and KO mice, we incubated whole explanted adrenal glands in vitro. The general microscopic structure of adrenal glands from both genotypes was similar (Figure S1). From each mouse, we incubated the two adrenal glands separately (Figure 1A). After an initial phase in isotonic conditions, we changed the conditions for one gland to be exposed to a hypotonic environment while the other stayed under isotonic conditions. We observed that hypoosmolar conditions increased aldosterone production in WT but not KO mice (Figure 1B, top). Neither genotype showed significant changes when the extracellular osmolality was held constant at 312 mosmol/kg H_2_O (Figure 1B, bottom).

### 2.2 Loss of ClC‐2 Prevents Osmolality‐Induced Increases of Intracellular Calcium

**FIGURE 2:**
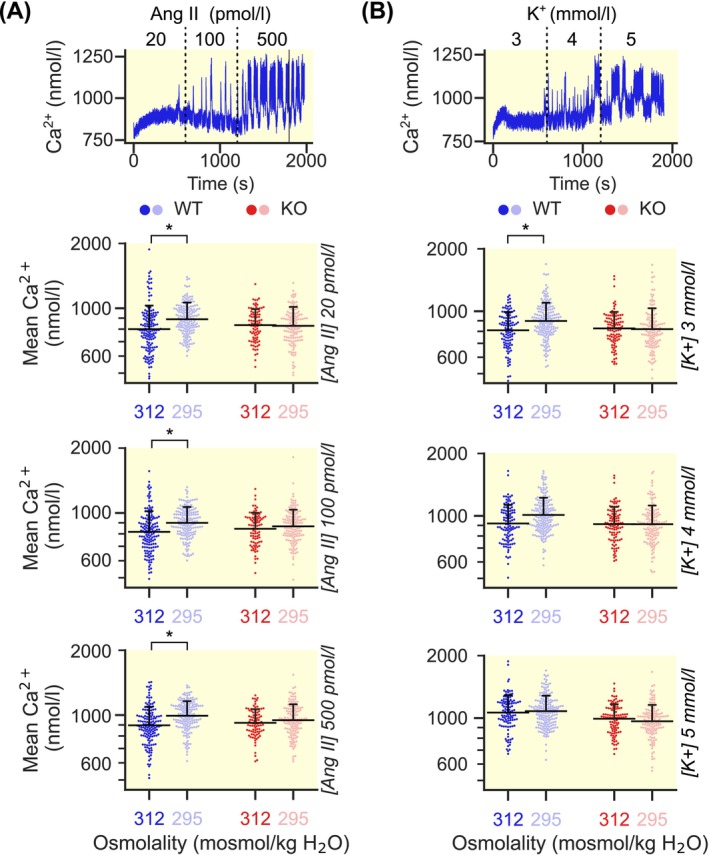
Absolute intracellular calcium concentrations increase under some hypotonic conditions in WT but not KO. (A) Top: Representative WT recording of a single ZG cell (at 312 mosmol/kg H_2_O) demonstrating the course of the experiment. Ang II was perfused in increasing concentrations for approximately 10 min each while extracellular K^+^ was kept constant at 4 mmol/L. Bottom: Absolute intracellular calcium concentrations at three different stimulations with Ang II (shown in italic font) and either isotonic (312 mosmol/kg H_2_O, dark colors) or hypotonic conditions (295 mosmol/kg H_2_O, light colors). In the ZG from WT mice (blue), the intracellular calcium concentration increased with hypotonicity while it remained unaffected in cells from KO mice (red). (p_WT,20AngII_ = 0.02, *; p_WT,100AngII_ = 0.03; *p_WT,500AngII_ = 0.01, *; p_KO,20AngII_ = 0.59; p_KO,100AngII_ = 0.51; p_KO,500AngII_ = 0.58; likelihood ratio tests of linear mixed models; further information in Table S1). Each dot represents one cell, horizontal black lines indicate the mean, with the whisker representing the SD (only the positive whisker is shown for clarity). (B) Top: Representative WT recording of a single ZG cell (at 312 mosmol/kg H_2_O) demonstrating the course of the experiment. K^+^ was perfused in increasing concentrations for approximately 10 min each while Ang II was kept constant at 100 pmol/L. Bottom: Absolute intracellular calcium concentrations at three different concentrations of extracellular K^+^ (shown in italic font). In the ZG from WT mice (blue), the intracellular calcium concentration only increased significantly at 3 mmol/L of K^+^ and remained unaffected in cells from KO mice (red). (p_WT,3K+_ = 0.03, *; p_WT,4K+_ = 0.07; p_WT,5K+_ = 0.46; p_KO,3K+_ = 0.98; p_KO, 4K+_ = 0.82; p_KO, 5K+_ = 0.66; likelihood ratio test of linear mixed model; further information in Table S2). Each dot represents one cell, horizontal black lines indicate the mean, with the whisker representing the SD (only the positive whisker is shown for clarity).

Increased aldosterone synthesis usually follows an increase of mean intracellular calcium concentrations over time. Using ratiometric Fura‐2 imaging, we found that WT ZG cells indeed showed significantly higher intracellular calcium concentrations when exposed to hypotonic solutions for different concentrations of Ang II (Figure 2A). This effect was less prominent in the presence of high extracellular K^+^ concentrations (Figure 2B). In both genotypes, increasing stimulus intensity resulted in proportional increases of intracellular calcium concentrations (Tables S1 and S2).

In contrast, cells from KO mice did not respond to changes in extracellular osmolality regardless of stimulation with Ang II (Figure 2A) or K^+^ (Figure 2B).

Following an acute change of extracellular osmolality, both WT and KO mice showed an immediate rise in calcium activity followed by a strong decrease below baseline levels in WT after approximately 15 min. This contrasted with the activity in KO mice where spiking remained elevated in comparison (Figure 2A). Closer analysis showed unchanged Ang II‐dependent spiking between genotypes (Figure 2B) but revealed a leftward shift in potassium‐dependent activation of calcium spiking in KO mice (Figure 2C).

Taken together, these results suggest that lower calcium concentrations in the KO are not due to lower spiking frequencies.

### 2.3 ClC‐2 Mediates Chloride Efflux Under Hypoosmolar Conditions

**FIGURE 3:**
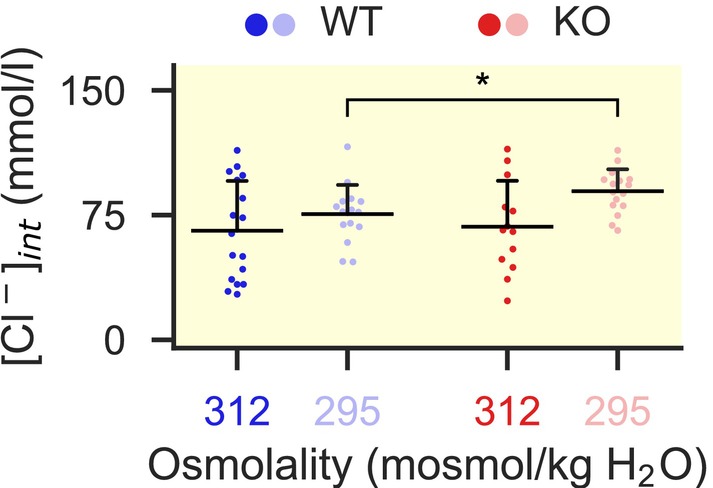
Extracellular hypoosmolality leads to increases of intracellular chloride in *Clcn2* KO mice. Chloride concentrations for the genotypes and conditions indicated. (All values mean ± SD: 312 mosmol/kg H_2_O, WT: 65.5 ± 29.1 mM, *n* = 17 recordings from 6 mice; KO: 68.0 ± 26.5 mM, *n* = 13 recordings from 5 mice; *p* = 0.81, *t* = −0.23; 295 mosmol/kg H_2_O: WT: 75.6 ± 16.9 mM, *n* = 15 recordings from 5 mice; KO: 89.3 ± 12.8 mM, *n* = 16 recordings from 5 mice; *p* = 0.019, *, *t* = −2.48; Student's *t*‐test). Each dot represents one recording, horizontal black lines indicate the mean, with the whisker representing the SD (only the positive whisker is shown for clarity).

To test whether ClC‐2 mediates net chloride efflux under hypoosmolality, we measured intracellular chloride concentrations in ZG cells of acute murine adrenal gland slices using the fluorescent indicator MEQ (Figure 3). We confirmed that the MEQ signal rises in a linear fashion with increasing intracellular chloride concentrations between 0 and 150 mM (Figure 3B). For each slice, we converted measured signals in individual cells to absolute chloride concentrations using a 2‐point calibration after each recording (Figure 3A).

Under isotonic conditions, both genotypes showed similar baseline chloride concentrations (Figure 3). In contrast, during hypotonic exposure (295 mosmol/kg H_2_O), KO cells presented higher steady‐state chloride concentrations than WT cells, suggesting that ClC‐2 prevents chloride accumulation during extended periods of hypotonicity.

### 2.4 Systemic Hypoosmolality Increases Aldosterone Production

To test whether ClC‐2's role in osmo‐sensing affects aldosterone production in vivo, we induced chronic hypoosmolality in WT mice using desmopressin. This synthetic analog of vasopressin activates V2 receptors in the kidney, resulting in excessive water reabsorption and creating a hyponatremic hypoosmolality. For continuous release, we surgically implanted osmo‐pumps that slowly released desmopressin over several days.

**FIGURE 4:**
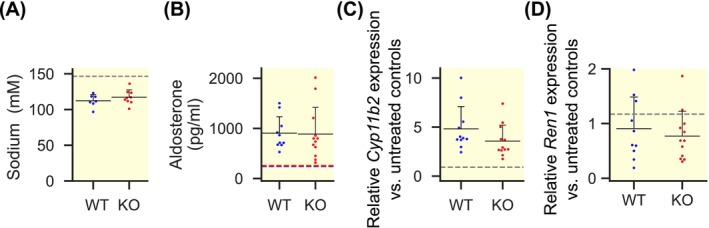
Aldosterone production under hyponatremic hypoosmolality is similarly increased in *Clcn2* KO compared to WT mice. Each dot represents data from one gland, horizontal black lines indicate the mean, with the whisker representing the SD (only the positive whisker is shown for clarity). (A) Plasma sodium concentrations were not different between genotypes (mean ± SD; WT: 112.3 ± 8.6 mM, *n* = 7 mice; KO: 117.2 ± 10.1 mM, *n* = 9 mice; *p* = 0.32, *t* = −1.03, Student's *t*‐test) but were significantly lower than in previously published mice with a similar background (gray dashed line; see Discussion for a detailed comparison). (B) Mean plasma aldosterone concentration after 1 week of forced hyponatremia. WT and KO values were similar (mean ± SD, WT: 904 ± 327 pg/mL, *n* = 10 mice; KO: 886.74 ± 532 pg/mL, *n* = 12 mice, *p* = 0.57, U = 69; Mann–Whitney‐U test). Aldosterone concentrations were, however, higher than in untreated controls reported previously [25] (WT, blue: 243 pg/mL; KO, red: 255 pg/mL; both shown as dashed lines for comparison). (C) The relative expression of aldosterone synthase (*Cyp11b2*) in the adrenal gland was significantly higher than in untreated controls (mean ± SD, untreated C57BL/6: 0.90 ± 0.48 (mean shown as dashed line for comparison), *n* = 8 mice; WT: 4.82 ± 2.28, *n* = 11 mice, versus untreated: *p* < 0.001, U = 0; KO: 3.57 ± 1.63, *n* = 12 mice; versus untreated: *p* < 0.001, U = 1; Mann–Whitney‐U test). However, while the mean values suggest less *Cyp11b2* expression in KO, this difference was not statistically significant (KO versus WT: *p* = 0.069, U = 36; Mann–Whitney‐U test). (D) The relative expression of renin (*Ren1*) was similar in WT and KO kidneys compared to untreated controls (mean ± SD: Untreated C57BL/6: 1.17 ± 0.86, *n* = 11 (mean is shown as dashed line for comparison); WT: 0.91 ± 0.58, *n* = 10 mice, versus untreated: *p* = 0.46, *U* = 66; KO: 0.77 ± 0.45, *n* = 12 mice; versus untreated: *p* = 0.281, *U* = 84; Mann–Whitney‐*U* test). There was no statistical difference between implanted WT and KO mice (*p* = 0.668, *U* = 53; Mann–Whitney‐*U* test).

Both genotypes showed similar degrees of hyponatremia (Figure 4A) with concurrently high aldosterone levels (Figure 4B).

The expression of aldosterone synthase (*Cyp11b2*) in adrenal glands appeared slightly higher in WT than in KO, but the difference was not statistically significant (Figure 4C). Renin (*Ren1*) expression in kidneys was highly variable with no appreciable difference between the two genotypes and no increase over untreated controls (Figure 4D).

## 3 Discussion

Studies using perfused whole canine adrenal glands have demonstrated that even small decreases in osmolality amplify aldosterone secretion [34]. Under physiological conditions, this appears to be mediated by changes to calcium spiking as for other stimuli [35]. The increase in aldosterone synthesis was previously found to be chloride‐dependent [33], but the molecular mechanisms remain unclear.

We here link this mechanism to the ClC‐2 chloride channel in vitro. Given the reported osmo‐sensitivity of this channel [13], we hypothesized that hypoosmolality may serve as its activating stimulus.

In agreement, we observed an increase of aldosterone production under hypoosmolar conditions in WT but not KO adrenal glands (Figure 1). Within the ZG, mean intracellular calcium concentrations increased in WT mice, while no such effect was observed in KO cells during exposure to hypotonic conditions (Figure 2).

**FIGURE 5:**
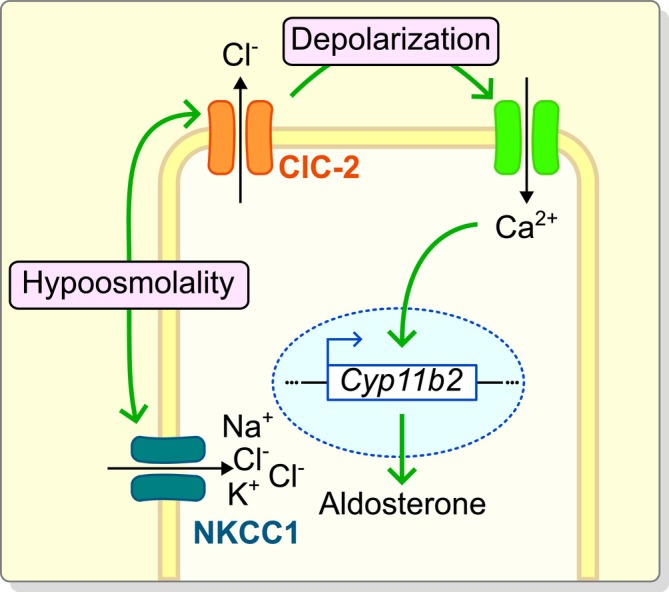
Schema of the role of ClC‐2 in the ZG response to hypoosmolality. ClC‐2 is activated by hypoosmolality, which leads to a depolarizing chloride efflux. This serves as a recycling pathway for chloride influx via NKCC1. The induced depolarization facilitates calcium influx, which increases aldosterone production, most prominently through the upregulation of the transcription of aldosterone synthase (Gene: *Cyp11b2*).

The high levels of intracellular chloride in the ZG [10] (Figure 3) combined with the known hyperpolarized resting membrane potential [3] suggest efflux of chloride from the ZG as a primary function of ClC‐2. In agreement, we observed lower chloride concentrations in the ZG of WT compared to *Clcn2* KO mice under sustained hypoosmolar conditions (Figure 3). A chloride efflux through ClC‐2 should depolarize ZG cells, which we infer activates voltage‐gated calcium channels [25, 26], explaining the observed differences in intracellular calcium concentrations and subsequently aldosterone production (Figure 5).

Furthermore, it has recently been shown that the effects of hypoosmolality on ZG aldosterone production are attenuated by inhibiting the sodium‐potassium‐2 chloride co‐transporter NKCC1 [35]. If NKCC1 does play a significant role in the ZG response to hypoosmolality, its activity would result in an increase of intracellular chloride, facilitating the depolarizing effect of ClC‐2 and the influx of calcium via voltage‐gated calcium channels (Figure 5).

For intracellular chloride concentrations of around 70 mM, the Nernst potential for chloride is about −10 mV. However, the actual depolarization caused by the activation of ClC‐2 is likely much smaller, owing to their small conductance and the high expression of potassium channels. It is therefore conceivable that the depolarizing effect of ClC‐2 under hypoosmolar conditions is small enough to activate voltage‐gated calcium channels within the range of their window currents, leading to constitutive increases of ZG calcium without increases in spiking activity (Figure 2A; Figure S2).

In the physiologic ZG, calcium concentrations appear to be regulated by increasing calcium spiking in response to the primary stimuli, Ang II and K^+^ [4, 25, 35, 36] (Figure S2). However, under hypoosmolality, ZG cells in slices from KO mice showed an increase in spiking activity despite lower cytosolic Ca^2+^ relative to WT (Figure S2; Figure 2). This effect may be due to the activation of calcium‐dependent potassium channels in the WT as previously suggested for mice carrying a mutation in the Ca_V_3.2 calcium channel [37]. An important implication of this finding is that, while intracellular Ca^2+^ concentrations and spiking frequency may be linked under most physiological conditions, absolute calcium concentrations may be more important for the regulation of aldosterone production than spiking frequencies.

Upon a hypoosmolar challenge, cells initially quickly extrude ions, particularly chloride, to counteract cell swelling in a process called rapid volume decrease (RVD) [38]. RVD is mediated by the activation of several ion conductances [38, 39]. In our experiments, this phase likely corresponds to the sharp initial increase in calcium spiking seen in the first few minutes. During this phase, ClC‐2 apparently only plays a minor role as seen by the strong increase in spiking that was not significantly different between WT and KO (Figure S2A).

Subsequent long‐term osmotic adaptation requires the slower efflux of other, non‐ionic substances (osmolytes). This allows for the re‐entry of ions and restoration of necessary ion balances for cellular function [39]. In our experiments, this period likely starts after the initial peak, on a time frame similar to other cell types [40, 41]. Given the increased [Cl^−^]_int_ in KO mice (Figure 3), it is conceivable that ClC‐2 serves as a chloride recycling pathway for other ion transporters during this phase, such as the aforementioned NKCC1 [26, 35], facilitating the re‐entry of Na^+^ and K^+^ [42]. Our hypothesis is further supported by the observation that intracellular chloride concentrations show less variability under hypoosmolar than isotonic conditions (Figure 3). This is likely due to secondary active transport of chloride compared to a more passive distribution during isotonic conditions.

Overall, ClC‐2 in the murine adrenal gland may further serve to sensitize the ZG for Ang II and thus, upstream, to renin during phases of prolonged hypoosmolality. Such sensitization reduces the necessity for continuous renin synthesis and release during phases of prolonged hypoosmolality.

While our study was conducted in mice, ClC‐2 is also expressed in human adrenal glands [10] and should have similar functions there. Our slice‐based approach cannot capture all aspects of integrated organ function as demonstrated in perfused whole‐gland studies [32], yet it enables detailed investigation of cellular mechanisms and ion fluxes that would be difficult to measure in intact organs. Direct determination of the predicted depolarization due to the activation of ClC‐2 via current clamp recordings was not feasible. Whole cell and perforated patch recordings clamp (some of) the intracellular ion concentrations, precluding the dynamic changes in response to changes in the osmotic gradient. On cell or sharp electrode recordings suffer from worse resolution and are not feasible due to the significant movement of the slice during swelling and shrinking. The use of fluorescent voltage‐indicators might be a promising strategy but the use of several dyes (FluoVolt, Di‐4‐ANEPPS or RH795) did not result in measurable signals in our hands.

In vivo, the effect of hypoosmolality on aldosterone production apparently does not solely depend on ClC‐2. Upon administration of desmopressin in combination with a low‐sodium, fluid‐rich diet, we generated a significant hyponatremia compared to previously published data (mean ± SD of untreated WT taken from [43]; 146.6 ± 2.3 mM, *n* = 20, vs. implanted WT: *p* = 2.15 × 10^−15^, *t* = −16.7; vs. implanted KO: *p* = 4.5 × 10^−13^, *t* = −12.5; Student's *t*‐test with Bonferroni correction). Aldosterone levels increased in both WT and KO animals, as did levels of *Cyp11b2* expression, despite unchanged levels of the upstream regulator renin in the kidney (*Ren1*; Figure 4). This suggests mechanisms other than the renin‐angiotensin system controlling aldosterone production. A potential explanation for this is stress‐induced secretion of adrenocorticotrophic hormone (ACTH) after surgery which may induce aldosterone secretion particularly at low levels of systemic sodium [44]. Although we did not measure ACTH in our animals, we surmise that ACTH stimulation may have overridden genotype‐dependent effects on aldosterone production—aldosterone levels and *Cyp11b2* expression were slightly, though not significantly, lower in KO animals. However, statistical power to detect such a small effect was only about 40% (*post hoc* analysis), suggesting that much larger sample sizes would be required to further investigate the in vivo role of ClC‐2 in hypoosmolality‐induced aldosterone production. Milder hyponatremia and hypoosmolality with less stress might be useful in this setting. Of note, in vivo, hypoosmolality was achieved through hyponatremia, unlike in our ex vivo experiments, in which sodium concentrations remained constant.

In summary, we have found a role of ClC‐2 mediated chloride efflux in the maintenance of chloride homeostasis in the ZG in response to hypoosmolality. This may at least partially explain the known chloride dependence of the hypoosmolality‐induced aldosterone synthesis, but more studies on the exquisite sensitivity of aldosterone production to changes in osmolality are needed to fully understand the involved mechanisms.

## 4 Methods

### 4.1 Good Publication Practice

The experiments, their analysis and their reporting follow the good publication practice in physiology [45] and the “Essential 10” of the ARRIVE 2.0 guidelines [46].

### 4.2 Breeding *of Clcn2* KO Mice

A single ClC‐2‐deficient (*Clcn2* KO) mouse was originally provided by Thomas Jentsch (FMP Berlin) [21]. Breeding was performed in the Forschungseinrichtungen für Experimentelle Medizin at Charité—Universitätsmedizin Berlin. The colony was generated by backcrossing with C57BL/6NCrl mice. As male homozygous KO mice are infertile [21], heterozygous males were bred with homozygous KO females. WT controls stemmed from the same colony but had to be bred separately.

All mice were housed in a 12 h light/dark cycle (6 a.m. to 6 p.m) with unrestricted access to food and water, environmental enrichment and under specific‐pathogen free conditions. All animal experiments conducted in this study were performed in compliance with German animal welfare laws and directives, as well as institutional guidelines and the 3R principle. The protocols for animal experiments were approved (G210/17) and the use of organs for scientific purposes following euthanasia registered (T0425‐17 and T‐CH0010/23) by the relevant authorities (Landesamt für Gesundheit und Soziales, LaGeSo, Berlin and Charité—Universitätsmedizin Berlin) as required.

### 4.3 Induction of Hypoosmolality in Mice

Hypoosmolality was induced via the chronic release of desmopressin from osmotic pumps [47, 48]. Sample sizes were calculated based on previously reported resting aldosterone levels [49]. Thirty mice per genotype were considered necessary to detect a 15% difference in blood aldosterone levels with 80% power. However, an intermediate analysis of means and variances during our experiments revealed much larger values and thus much higher required sample sizes than expected (more than 90 per genotype). It was therefore decided to terminate the experiments early after the number of mice shown (11 WT mice, 18 KO mice).

Mice included in the analysis were between 10 and 14 weeks old, with 11 WT (5 male, 6 female) and 12 KO mice (5 male, 7 female) being successfully implanted.

An overview of the experimental animals included and excluded during each step of the procedure can be found as Figure S4.

Osmotic pumps (ALZET, Model 1004) were filled with desmopressin acetate (0.002 mg/mL Minirin prepared in sterile 0.9% NaCl) under a sterile workbench according to the manufacturer's instructions. Before implantation, the filled pumps were activated in sterile 0.9% NaCl solution at 37°C for 48 h.

Surgery was performed in random order of cages in respect to the genotype (determined by their position in the rack) and by the same personnel. Mice were anesthetized using isoflurane (induction chamber: oxygen flow of 1 L/min 100% oxygen, 5 Vol.‐% isoflurane; continuous anesthesia under an inhalation mask: oxygen flow of 1 L/min 100% oxygen, 2–3 Vol.‐% isoflurane on top of a heating blanket (37°C)). Carprofen (5 mg/kg) was injected subcutaneously prior to the procedure for analgesia.

A skin incision of about 0.5–1 cm was made between the scapulae, and a small pocket was formed using blunt scissors. The pumps were inserted with the flow moderator pointing away from the incision. The incision was then closed with a wound closure system (autoclip), and each mouse was monitored until it was fully awake.

After implantation, mice were housed in regular home cages for 5 days. Water and food were provided ad libitum. Food was provided as a low‐sodium gel (36.9 mg sodium per 100 g gel; AIN76A diet gel, ClearH_2_O; exchanged daily) to support the induction of hypoosmolality by combining sodium and water uptake.

Afterwards, blood, urine, and organs were collected during the early afternoon (again randomized per cage as described above for the surgical procedure) following i.p. anesthesia (ketamine, 100 mg/kg and xylazine, 10 mg/kg) and terminal blood collection via cardiac puncture. The blood was collected into Microtainer K2‐EDTA tubes (Becton Dickinson) and Lithium‐Heparin tubes (Becton Dickinson). All blood samples were centrifuged (10 min, 2000 g, 4°C), and the supernatant (plasma) was stored at −20°C until further analysis.

The aldosterone plasma levels were analyzed using an ELISA (RE52301, IBL International GmbH). Blood plasma sodium was measured using an AU480 Clinical Chemistry Analyzer (Beckman Coulter) at the Animal Phenotyping Facility at the Max‐Delbrück‐Centrum Berlin. No attempt at blinding in regard to the genotype was made.

### 4.4 Aldosterone Production of Explanted Adrenal Glands

The explanted adrenal glands were carefully cut by hand into four equal‐sized pieces and stored in carbogen‐gassed, bicarbonate buffered solution (BBS_Prep,Supp_) at 312 mosmol/L kg H_2_O supplemented with 2 mmol/L CaCl_2_ (Table 1) in a cell culture insert in a 12‐well plate (pore diameter: 1 μm, Greiner Bio‐One). The well and the inside of the cell insert were each filled with 1000 μL BBS_312,5K_^+^ (Table 1), supplemented with 100 pmol/L Ang II (1 mmol/L stock solution prepared in H_2_O every month; A9525, Sigma Aldrich) and continuously gassed with carbogen at RT. After 30 min, 500 μL of the solution (outside of the insert) was collected (sample t_0_). Since two adrenal glands were collected from each animal, the solutions were exchanged in one well and insert with fresh BBS_5K_^+^_,312_ and in the other with BBS_295.5K_^+^ solution, supplemented with 100 pmol/L Ang II. After 90 min, solutions were again exchanged for fresh solutions of the same osmolality and 500 μL were collected after another 30 min. The aldosterone concentrations were measured using ELISA (RE52301, IBL International GmbH) according to the manufacturer's instructions.

**TABLE 1:** Composition of the “Bicarbonate Buffered Solutions” (BBS) used in this study.

Compound in mmol/L	BBS_Prep_	BBS_Prep,Supp_	BBS_295,2K_^+^	BBS_295,7K_^+^
NaCl	100	100	100	100
KCl	2	2	2	2
NaHCO_3_	26	26	26	26
CaCl_2_	0.1	2	2	2
MgCl_2_	5	5	1	1
NaGluconate	—	—	5	—
KGluconate	—	—	—	5
D‐Glucose	10	10	10	10
HEPES	10	10	10	10
Osmolality (mosmol/kg H_2_O)	291.3	297	295	295
For isotonic solutions (~312 mosmol/kg H_2_O)				
Mannitol	+17	+17	+17	+17
For hypertonic solution (~337 mosmol/kg H_2_O)				
Mannitol	+42	+42	+42	+42

### 4.5 Slice Preparation

Mice were anesthetized by using isoflurane (400 μL as an open drop in a 2 L beaker) and subsequently euthanized by cervical dislocation. Both adrenal glands were removed from the mice via the abdominal cavity. Any remaining fat surrounding the adrenal glands was carefully removed. Afterwards, the adrenal glands were transferred into ice‐cold bicarbonate‐buffered solution (BBS_Prep_), which was continuously gassed with carbogen (95% O_2_ + 5% CO_2_). The osmolality of all solutions was adjusted to the desired osmolality (Table 1) at the start of the respective experiment.

Before the adrenal glands were cut in a vibratome at 120 μm thickness (7000 smz‐2, Campden Instruments), they were embedded in 4% low‐melting agarose. During the slicing process, the organ and slices were stored in ice‐cold BBS_Prep_ solution, continuously gassed with carbogen. Afterwards, slices were transferred into a carbogen gassed BBS_Prep_ at 35°C for 15 min and then stored for up to 6 h at RT in carbogen gassed BBS_Prep,Supp_ supplemented with 2 mmol/L CaCl_2_.

### 4.6 Preparation of diH‐MEQ

For Cl^−^ imaging experiments in living cells, MEQ was reduced to the cell‐permeable form diH‐MEQ. This synthesis was performed according to the manufacturer's instructions. All steps were performed while purging oxygen from the reaction using a slow stream of nitrogen. In detail, diH‐MEQ was prepared from 5 mg of MEQ (21 250, AAT Bioquest) and dissolved in 0.1 mL of distilled water. During the dissolution process, a small amount of (20–50 μL) of a 12% (120 mg/mL) aqueous solution of sodium borohydride (424270010, Thermo Scientific Chemicals) was prepared for immediate use. 10 μL (32 μmol) of the sodium borohydride solution was slowly added. After 30 min, the yellow diH‐MEQ was extracted from the solution with anhydrous ethyl acetate (270989‐250ML, Sigma‐Aldrich). The upper organic layer containing diH‐MEQ was transferred into a 4 mL sample vial (WHEATON amber with cap, Merck). The extraction step was repeated twice with an additional 0.5 mL of ethyl acetate each time. After the organic extracts were combined, 150 mg anhydrous sodium sulfate (S6547‐500G, Sigma‐Aldrich) was added to the vial and gently mixed to dry the organic layers and transferred to a 5 mL Schlenk sample tube (SP Wilmad‐LabGlass, VWR). The organic solvent was now carefully evaporated under vacuum in a water bath at 35°C–45°C. The solid diH‐MEQ (yellowish‐light green oil) was dissolved in 100 μL DMSO/10% Pluronic F‐127 (~150 mmol/L) by vortexing thoroughly for 30 s. 2 μL aliquots were prepared and transferred into 1.5 mL screw cap microtubes that were tightly sealed under N_2_ and stored at −80°C for use within 1 week.

### 4.7 Staining of Adrenal Slices With Calbryte 520 AM, Fura‐2 AM or diH‐MEQ

For staining the slices, a cell culture insert (pore diameter: 1 μm, Greiner Bio‐One) was added to a single well of a 24‐well plate. The well (outside of the insert) was filled with 750 μL BBS_295,2K_^+^ and continuously gassed with carbogen. The insert was filled with 250 μL of BBS_295,2K_^+^ containing initially either 64 μmol/L Fura‐2 AM (21 023, AAT Bioquest; final well concentration: 16 μmol/L) and 0.16% Pluronic F‐127 or 37 μmol/L Calbryte 520 AM (20 650, AAT Bioquest; final well concentration: 9.25 μmol/L) and 0.001% Pluronic F‐127. For diH‐MEQ, the insert was filled with 250 μL containing initially either 600 μmol/L diH‐MEQ (final well concentration: 150 μmol/L) and 0.4% Pluronic F‐127. Regardless of the dye used, slices were left in the dyeing chamber for 1 h at RT in the dark.

### 4.8 Measurement of Ca^2+^ Signals Using Calbryte 520 AM

Calbryte 520 stained slices were transferred into the recording chamber of the microscope (Scientifica SliceScope). The stained slices in the recording chamber were continuously perfused with carbogen‐gassed solutions, heated by an inline heating coil to 30°C ± 1°C. For Calbryte 520 measurements, a blue LED (Channel 2B, pE‐300 ultra, CoolLED) passing through a 474/27 nm bandpass filter (AHF Analysentechnik) was used to excite the dye. Signal emissions were collected by a 40×/0.8 NA objective (LUMPlanFL N, Olympus), filtered through a 432/523/702 nm triple‐band filter (AHF Analysentechnik) and recorded using a sCMOS camera (OptiMOS, QImaging) every 100 ms with a 10 ms exposure.

### 4.9 Measurement of Absolute [Ca^2+^]_*i*_ Using Fura‐2 AM

Fura‐2 stained slices were transferred into the recoding chamber of the microscope (Scientifica SliceScope). The stained slices in the recoding chamber were continuously perfused with carbogen‐gassed solutions. For Fura‐2 measurements, a light source with 340 and 385 nm LEDs (FuraLED, Cairn Research) was used. Emission light was collected using a 40×/0.8 NA objective (LUMPlanFL N, Olympus), a 510/84 nm bandpass filter (AHF Analysentechnik) and a camera (OptiMOS, QImaging). Every 100 ms, two images with 10 ms exposure each were taken during excitation with the 340 and 385 nm LED, respectively.

The calibration of Fura‐2 signals was performed as previously described, using a calibration kit (Calcium Calibration Buffer Kit, Thermo Scientific), to obtain the 340/385 nm ratios to the corresponding [Ca^2+^]_*i*_ in Fura‐2 stained adrenal gland slices.

### 4.10 Measurement of Cl^−^ Concentrations Using diH‐MEQ

diH‐MEQ stained slices were transferred into the recording chamber of the microscope (Axioskop 2 FS, Zeiss). Slices were kept in the perfused chamber for 15 min before the start of the first recording to ensure an even distribution of the MEQ dye.

For MEQ measurements, a 340 nm LED (Thorlabs) passing through a 340/26 nm bandpass filter (Edmund Optics) was used for excitation. Emissions were recorded using a 40×/0.75 NA objective (Achroplan, Zeiss), a 510/84 bandpass filter (Edmund Optics), and a camera (GS3‐U3‐51S5M‐C, FLIR) every 20s with a 50 ms exposure.

MEQ signals were initially tested for linearity between Cl^−^ concentrations of 0–150 mmol/L (Figure 3B). For the final experiments, MEQ signals were calibrated on the same slice after each recording using only two Cl^−^ concentrations (0–80 mmol/L Cl^−^) by incubation with standard solutions (BBS_312,5K_^+^ or BBS_295,5K_^+^) supplemented with 10 μmol/L nigericin (11 437, Cayman Chemicals) and 20 μmol/L tributyltin chloride (T50202‐100G, Sigma‐Aldrich).

### 4.11 Analysis of Fluorescence Microscopy

Microscopy recordings were taken using Micro‐Manager 2.0 [50] and processed using Fiji [51]. After manual selection of individual cells and export from Fiji, further analysis was performed using custom python scripts [4, 37].

### 4.12 Quantitative Real‐Time PCR

The extraction/isolation of total RNA from adrenal glands and kidneys stored in RNAlater (Sigma‐Aldrich) at −20°C was performed using the RNeasy Mini Kit (Qiagen). Reverse transcription was performed using the Quantitect RT Kit (Qiagen). For adrenal and kidney cDNA samples, the TaqMan real‐time PCR expression master mix (Applied Biosystems) was used with *Gapdh* (Mm99999915g1, housekeeping gene), *Cyp11b2* (Mm00515624m1), and *Ren1* (Mm02342887_mH) probes. The gene expression was evaluated relative to *Gapdh*, and the mean ΔCT of WT controls was expressed as 2^ΔΔCt^ (fold change).

### 4.13 Statistics

Data were statistically analyzed using custom Python and R scripts. In Python, the Scipy [52] library was used for analysis. In particular, the scipy.stats.shapiro function was used to assess normality. If normality was violated, the scipy.stats.mannwhitneyu or scipy.stats.wilcoxon functions were used for statistical comparisons; otherwise the scipy.stats.ttest_ind function was used as stated in the text. Calcium concentrations were analyzed using linear mixed models in R using the lme4 package [53].

## Author Contributions

Marina Volkert: Development of methodology, acquisition of data, analysis of data, preparation of figures, editing the manuscript. Hoang An Dinh: Acquisition of data, editing the manuscript. Ute I. Scholl: Conceptualization, preparation of figures, writing and editing the manuscript. Gabriel Stölting: Conceptualization, analysis of data, curation of data, supervision, writing and editing the manuscript.

## Funding

This research was funded by the Deutsche Forschungsgemeinschaft (STO 1260/1–1 for Gabriel Stölting) and Stiftung Charité (BIH_PRO_406 to Ute I. Scholl).

## Ethics Statement

All animal experiments conducted in this study were performed in compliance with German animal welfare laws and directives, as well as institutional guidelines and the 3R principle. The protocols for animal experiments were approved (G210/17) and the use of organs for scientific purposes following euthanasia registered (T0425‐17 and T‐CH0010/23) by the relevant authorities (Landesamt für Gesundheit und Soziales, LaGeSo, Berlin and Charité—Universitätsmedizin Berlin) as required.

## Conflicts of Interest

The authors declare no conflicts of interest.

## Supporting information


**Figure S1:** Hematoxylin and Eosin (HE) staining of formalin fixed, paraffin embedded (FFPE) adrenal gland slices.
**Figure S2:** ZG cells from Clcn2 KO mice show different adaptation of calcium signaling when exposed to hypoosmolality.
**Figure S3:** Calibration of MEQ fluorescence to Cl^−^ concentrations.
**Figure S4:** Flowchart showing the use of animals for the implantation of desmopressin filled osmopumps.
**Table S1:** Statistical information for the angiotensin II dependent calcium concentrations in ZG cells.
**Table S2:** Statistical information for the potassium dependent calcium concentrations in ZG cells.

## Data Availability

Raw microscopy video files can only be provided upon reasonable request and must be individually arranged due to their file sizes. All extracted data and scripts used to generate the analyses and figures presented within this manuscript are available at zenodo [54] (https://zenodo.org/records/15051779).
