# Supplementary material for: ClC‐2 Contributes to Hypotonicity‐Induced Adrenal Aldosterone Secretion
Source: Acta Physiol (Oxf). 2026 Jan 28;242(3):e70168. doi: 10.1111/apha.70168 (PMC12852535; doi:10.1111/apha.70168)
Supplement: Supplementary file 1 — Figure S1: Hematoxylin and Eosin (HE) staining of formalin fixed, paraffin embedded (FFPE) adrenal gland slices. Figure S2: ZG cells from Clcn2 KO mice show different adaptation of calcium signaling when exposed to hypoosmolality. Figure S3: Calibration of MEQ fluorescence to Cl− concentrations. Figure S4: Flowchart showing the use of animals for the implantation of desmopressin filled osmopumps. Table S1: Statistical information for the angiotensin II dependent calcium concentrations in ZG cells. Table S2: Statistical information for the potassium dependent calcium concentrations in ZG cells. [file APHA-242-e70168-s001.docx]

**Supplementary Material to:**

**ClC-2 contributes to hypotonicity-induced adrenal aldosterone secretion**

Marina Volkert^1^, Hoang An Dinh^1,2^, Ute I. Scholl^1,3^ and Gabriel Stölting^1^

^1^ Berlin Institute of Health at Charité – Universitätsmedizin Berlin, Center of Genomic Medicine, Berlin, Germany

^2^ Institute of Translational Physiology, Charité – Universitätsmedizin Berlin, Berlin, Germany

^3^ Department of Nephrology and Medical Intensive Care, Charité – Universitätsmedizin Berlin, Berlin, Germany

**
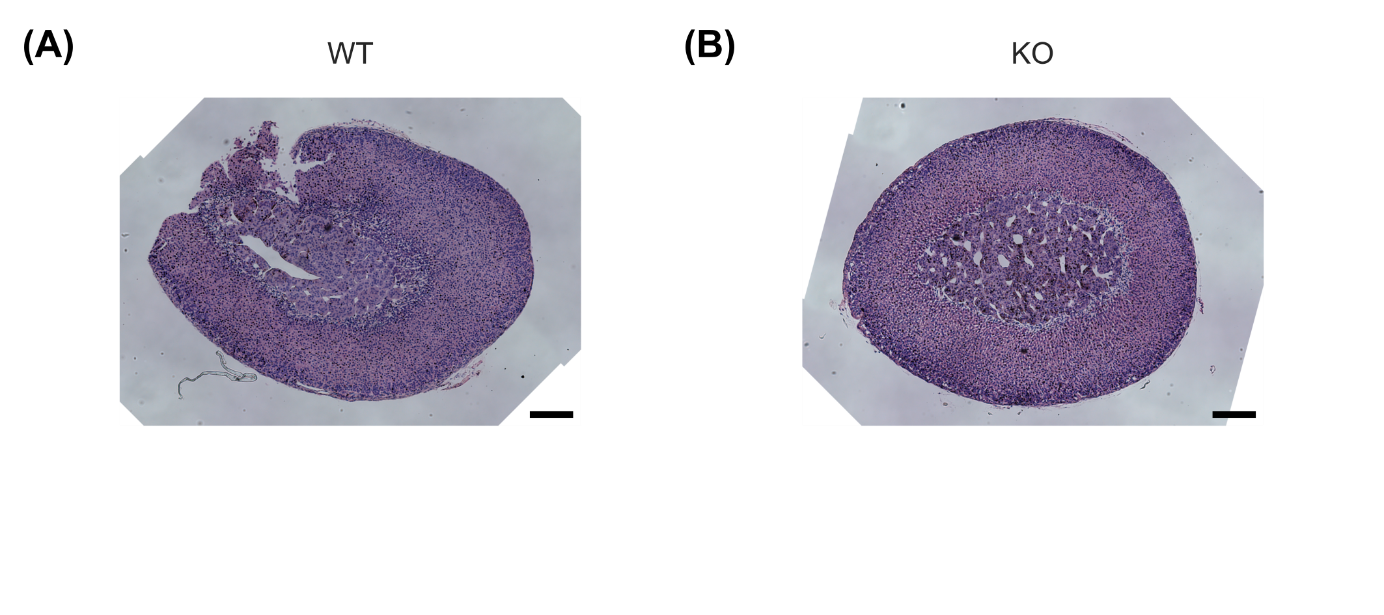
Supplementary Figure 1.** Hematoxylin and Eosin (HE) staining of formalin fixed, paraffin embedded (FFPE) adrenal gland slices. Representative images for a male WT **(A)** and a male KO **(B)**. Scale bars represent 200 µm. Images were digitally rotated for better comparison.


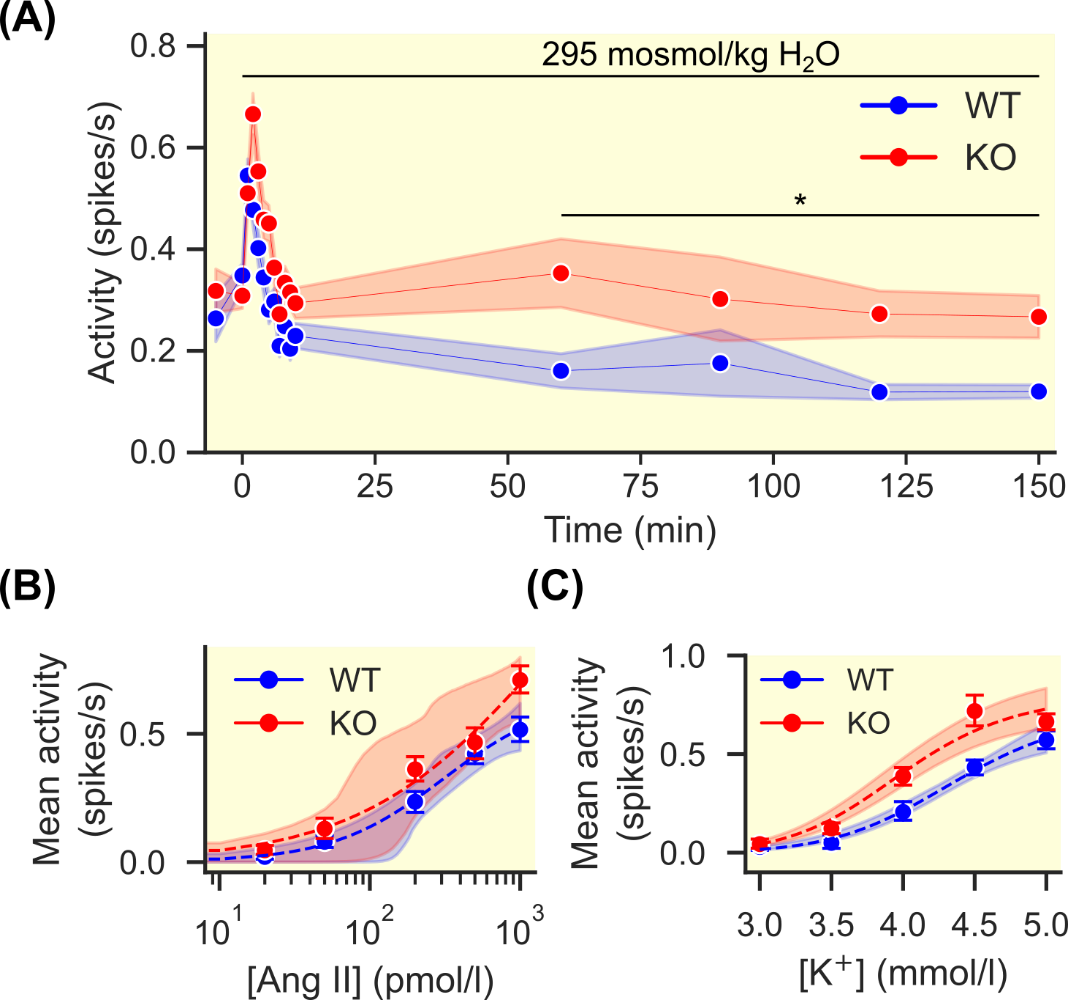


**Supplementary Figure 2.** ZG cells from *Clcn2* KO mice show different adaptation of calcium signaling when exposed to hypoosmolality.

**(A)** Changes in the spiking activity upon exposure to hypoosmolality starting from isotonic conditions (n_WT_ = 6 mice; n_KO_ = 6 mice). Immediately after the switch to a hypotonic environment (starting at t = 0 min), spiking activity rose in both genotypes. The slightly higher mean activity observed in KO mice during the first 10 minutes, which likely correspond to the RVD, was not significantly different to WT (p = 0.081, 𝒳^2^(1) = 3.25). During longer incubation, however, the activity in KO remained significantly elevated relative to WT cells (p = 0.009, 𝒳^2^(1) = 6.91). Recordings were performed in the presence of 100 pmol/l Ang II and 5 mmol/l K^+^ Statistical analysis was performed using a separate likelihood ratio tests of linear mixed models for the values before and after 10 minutes with genotype as fixed and the animal ID as random effects.

**(B)** Calcium spiking activity increases with extracellular Ang II concentrations (constant 4 mmol/l K^+^). There is no significant difference between ZG cells from WT and *Clcn2* KO mice at an osmolality of 295 mosmol/kg H_2_O. Dashed lines represent a fit with a Hill equation, the shaded areas comprise the 95% confidence intervals of all combined fits. Measured data is shown as mean (circles) with 95% CI of the measured data shown as errorbars. Fitting of the Hill equation was performed using a bootstrap algorithm with 10,000 resamples. WT (mean and 95% CI): V_max_ 0.65 (0.46-2.28), K_50_=311.31 (150.51-4626.54), n=1.16 (0.71-10.0); KO: V_max_ 1.59 (0.54-85.55), K_50_=1435.79 (65.35-2116015.40), n=0.71 (0.55-10.0).

**(C)** The potassium dependence of calcium spiking activity is shifted towards lower concentrations at an osmolality of 295 mosmol/kg H_2_O (constant 100 pmol/l Ang II). Dashed lines represent a fit with a Hill equation, the shaded areas comprise the 95% confidence intervals of all combined fits. Measured data is shown as mean (circles) with 95% CI of the measured data shown as errorbars. Fitting of the Hill equation was performed using a bootstrap algorithm with 10,000 resamples. WT (mean and 95% CI): V_max_ 0.73 (0.60-1.08), K_50_=4.37 (4.18-4.80), n=10.0 (7.47 -10.0); KO: V_max_ 0.81 (0.69-0.94), K_50_=3.99 (3.84-4.15), n=10.0 (9.31-10.0).


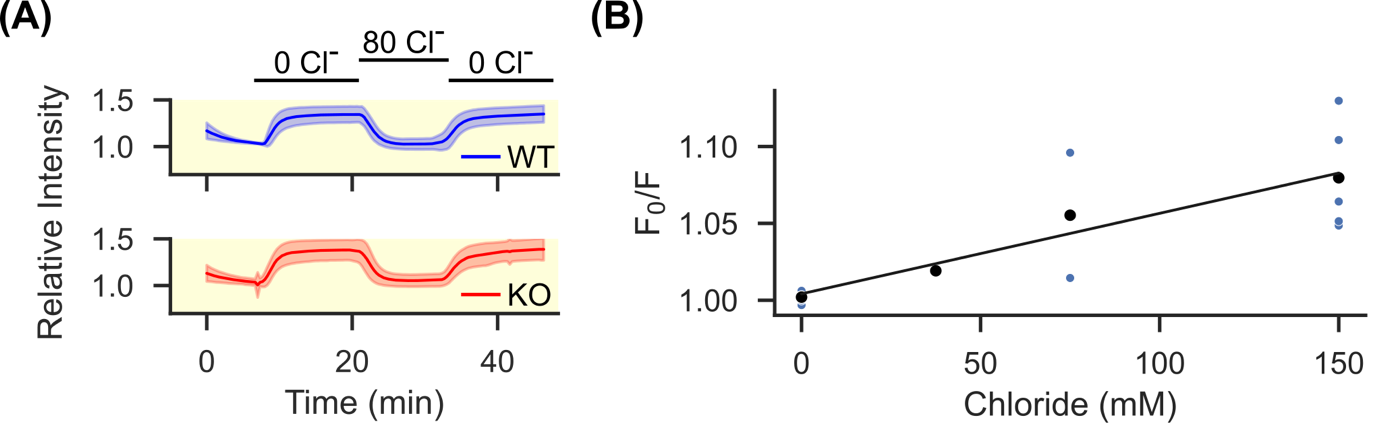


**Supplementary Figure 3.** Calibration of MEQ fluorescence to Cl^-^ concentrations.

**(A)** Average (thick line) ± SD (light area) of MEQ fluorescence traces at 295 mosmol/kg H_2_O. Initially, standard extracellular solution (constant 100 pmol/l Ang II and 5 mmol/l K^+^ ) was used and then exchanged for calibration solutions containing 0 or 80 mM Cl^-^ as indicated. All traces were individually normalized to the value prior to the first perfusion with 0 mM Cl^-^ for display. Steady-state values at the end of each perfusion condition were taken as representative of the chloride-dependent fluorescence for the indicated concentration. **(B)** Stern-Vollmer plot of the results of a separate calibration with 4 different chloride concentrations (0, 37.5, 75 and 150 mM) demonstrating a linear dependence of the MEQ signal Black circles represent mean values for each concentration, blue circles are from independent recordings (5 recordings from 3 mice). The black line is a linear fit of the individual data points.

**
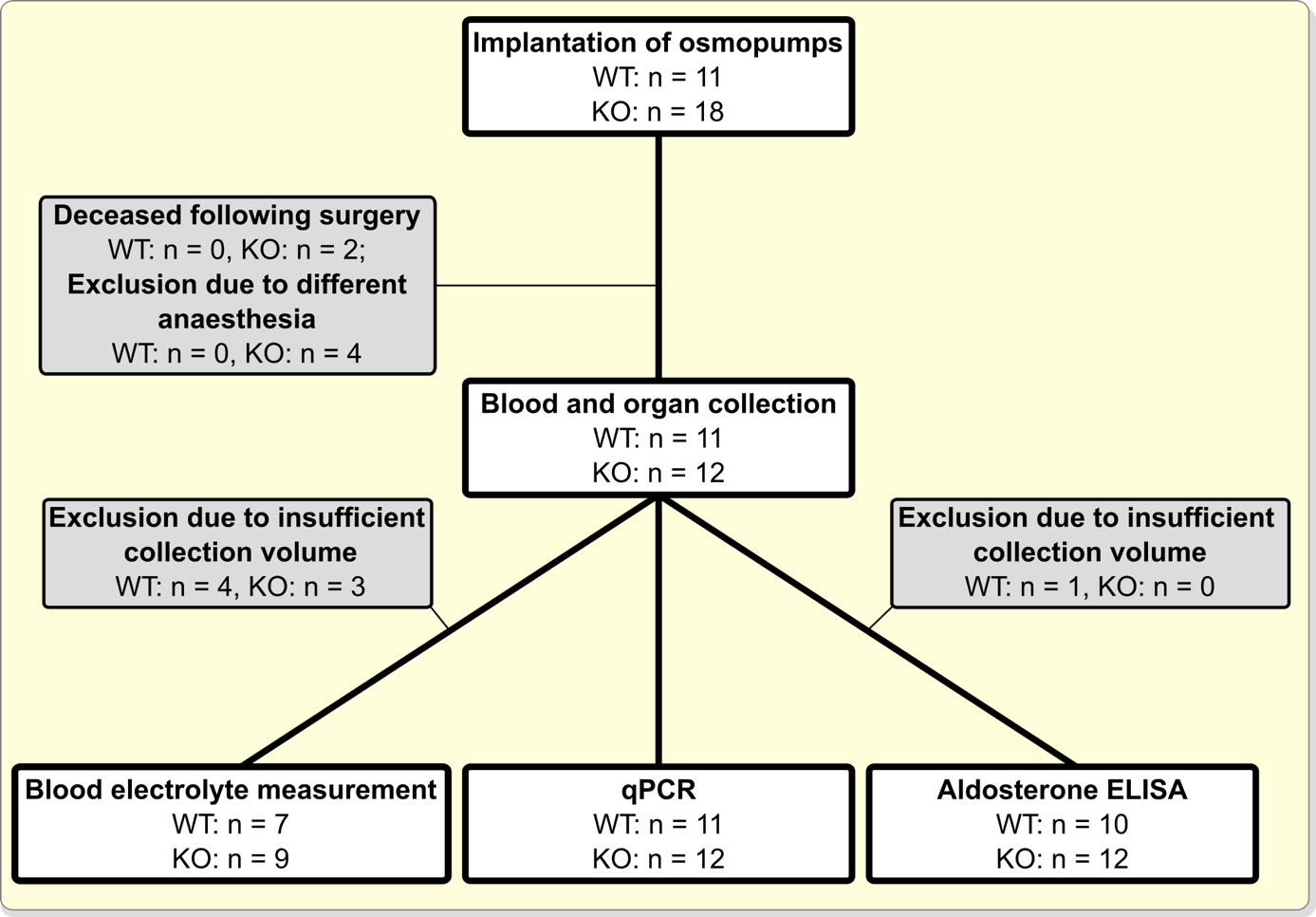
**

**Supplementary Figure 4.** Flowchart showing the use of animals for the implantation of desmopressin filled osmopumps. Due to the high mortality in the first batch of animals (2/6 died immediately after surgery), the method of anaesthesia was changed from ketamine/xylazine to isoflurane.

**Supplementary Table 1: Statistical information for the angiotensin II dependent calcium concentrations in ZG cells (Fig. 2A)**

|  |  |  |  | **[Ca^2+^]**  **(nmol/l)** | |  |  |
| --- | --- | --- | --- | --- | --- | --- | --- |
| **Genotype** | **Osmolality**  **(mosmol/kg H_2_O)** | **[Ang II]**  **(pmol/l)** | **N**  **(cells/**  **female mice/**  **male mice)** | **Mean** | **s.d.** | **P vs. 312** | **χ^2^**  **df=1** |
| **WT** | **295** | **20** | 169/2/2 | 903.04 | 157.54 | 0.02 | 5.08 |
|  |  | **100** | 169/2/2 | 911.85 | 148.25 | 0.03 | 4.98 |
|  |  | **500** | 169/2/2 | 1004.54 | 150.74 | 0.01 | 6.56 |
|  | **312** | **20** | 148/3/4 | 826.83 | 225.02 | - |  |
|  |  | **100** | 148/3/4 | 837.16 | 192.26 | - |  |
|  |  | **500** | 148/3/4 | 913.92 | 181.08 | - |  |
| **KO** | **295** | **20** | 134/3/3 | 846.15 | 185.54 | 0.59 | 0.29 |
|  |  | **100** | 134/3/3 | 880.43 | 165.54 | 0.51 | 0.44 |
|  |  | **500** | 134/3/3 | 960.37 | 163.50 | 0.58 | 0.30 |
|  | **312** | **20** | 90/3/4 | 846.93 | 143.05 | - |  |
|  |  | **100** | 90/3/4 | 856.18 | 144.09 | - |  |
|  |  | **500** | 90/3/4 | 930.15 | 133.25 | - |  |

**Supplementary Table 2: Statistical information for the potassium dependent calcium concentrations in ZG cells (Fig. 2B)**

|  |  |  |  | **[Ca^2+^]**  **(nmol/l)** | |  |  |
| --- | --- | --- | --- | --- | --- | --- | --- |
| **Genotype** | **Osmolality**  **(mosmol/kg H_2_O)** | **[K^+^]**  **(mmol/l)** | **N**  **(cells/**  **female mice/**  **male mice)** | **Mean** | **s.d.** | **P vs. 312** | **χ^2^**  **df=1** |
| **WT** | **295** | **3** | 182/2/2 | 915.76 | 188.08 | 0.03 | 4.68 |
|  |  | **4** | 182/2/2 | 1029.38 | 202.05 | 0.07 | 3.22 |
|  |  | **5** | 182/2/2 | 1096.04 | 191.20 | 0.44 | 0.60 |
|  | **312** | **3** | 109/3/4 | 825.79 | 160.90 | - |  |
|  |  | **4** | 109/3/4 | 939.18 | 195.14 | - |  |
|  |  | **5** | 109/3/4 | 1082.25 | 209.96 | - |  |
| **KO** | **295** | **3** | 133/3/2 | 842.68 | 208.73 | 0.98 | 8×10^-4^ |
|  |  | **4** | 133/3/2 | 932.02 | 198.88 | 0.82 | 0.05 |
|  |  | **5** | 133/3/2 | 980.86 | 178.63 | 0.66 | 0.20 |
|  | **312** | **3** | 103/2/3 | 840.36 | 160.37 | - |  |
|  |  | **4** | 103/2/3 | 929.59 | 181.68 | - |  |
|  |  | **5** | 103/2/3 | 1004.90 | 161.45 | - |  |
